# Modeling structure factor motivators behavior questionnaire: exploratory factor analysis

**Published:** 2019-08

**Authors:** Shila Hasanzadeh, Mohammad Asghari Jafarabadi, Homyoun Sadeghi-Bazargani

**Affiliations:** ^*a*^Tabriz University of Medical Sciences, Tabriz, Iran.

## Abstract

**Background:**

Exploratory Analysis Method is one of the most useful methods in all issues such as Traffic. The purpose of this study was to investigate the factor structure of the motorcyclist behavior questionnaire in relation to male motorcyclists in Tabriz city.

**Methods:**

For this purpose, 456 motorcyclists participated in this case-control study using a cluster sampling method to complete the questionnaire. This paper described the six stages of exploratory factor analysis (Identification of variables/ indicators expressing the subject, controlling fit of data to perform factor analysis, determine the method and the number of factors that must be extracted, select method rotation of factors, interpretation and naming factors, calculate factor scores) and the analytical decisions of each step have been proposed. Recommendations were presented for achieving the optimal results approach analysis subjects of the behavior motorcyclist.

**Results:**

The results were obtained by SPSS software output, which was used to measure the behavior of motorcyclist in the field of traffic, According to the Bartlett and KMO tests, the correlation and adequacy of the sample size, respectively, checking Scree Plot, 2 factors, Varimax rotation, and method principal axis factoring showed that distractions and the driver s mind conflicts as well as driving and high-speed travel by motorcycle compared to other motorcyclist behaviors most likely to cause an accident.

**Conclusions:**

The results of the study indicate the validity of motorist’s behavior questionnaire in the field of traffic. Intervention programs are highly recommended for those who drive a lot of mental disturbances and distractions and traveling with motorcycle.

**Keywords:**

Traffic, Exploratory Factor Analysis, MRBQ

